# Virome of Australia’s most endangered parrot in captivity evidenced of harboring hitherto unknown viruses

**DOI:** 10.1128/spectrum.03052-23

**Published:** 2023-12-04

**Authors:** Natalie Klukowski, Paul Eden, Muhammad Jasim Uddin, Subir Sarker

**Affiliations:** 1 Department of Microbiology, Anatomy, Physiology and Pharmacology, School of Agriculture, Biomedicine and Environment, La Trobe University, Melbourne, Victoria, Australia; 2 Wildlife Conservation and Science, Zoos Victoria, Werribee, Victoria, Australia; 3 School of Veterinary Medicine, Murdoch University, Murdoch, Western Australia, Australia; 4 Center for Biosecurity and One Health, Harry Butler Institute, Murdoch University, Murdoch, Western Australia, Australia; 5 Biomedical Sciences & Molecular Biology, College of Public Health, Medical and Veterinary Sciences, James Cook University, Townsville, Queensland, Australia; Changchun Veterinary Research Institute, Changchun, China

**Keywords:** orange-bellied parrot, *Neophema* birds, metagenomics, virome, evolution

## Abstract

The detection of emerging infectious diseases (EIDs) within endangered animal populations is increasing rapidly because of many factors, such as anthropogenic influences and climate change. Their impacts can be extensive and may further contribute to the processes risking the threat of extinction of these species. EIDs may be caused by a range of pathogens, including viruses, bacteria, parasites, and fungi. However, detection and diagnosis can be challenging with limited knowledge of existing pathogens in endangered wildlife. The critically endangered orange-bellied parrot (OBP, *Neophema chrysogaster*), with as few as 70 wild individuals, is a species at risk of extinctions, yet little research has been conducted into their existing viral diversity (virome). To determine the virome in a subset of the captive OBP population, this study characterized the fecal virome using a viral metagenomic approach. Analysis of the generated sequence data sets identified 11 viruses belonging to the families *Adenoviridae*, *Circoviridae*, *Parvoviridae*, and *Picornaviridae*. Strikingly, eight viruses were detected in the OBPs housed in Aviary 1 compared to only three viruses in OBPs housed at Aviary 2. In addition to detecting six novel viruses, this study also demonstrated ongoing infection with psittacine siadenovirus F. This study highlights the need to broaden this research to other populations of this species. Further virome characterization of co-habiting birds could also identify potential novel viruses and provide insight into their evolutionary relationship. These findings may contribute to strategic management and biosecurity plans for the conservation of endangered parrots.

**IMPORTANCE:**

The impact of circulating viruses on the critically endangered, orange-bellied parrot (OBP) population can be devastating. The OBP already faces numerous threats to its survival in the wild, including habitat loss, predation, and small population impacts. Conservation of the wild OBP population is heavily reliant on supplementation using OBPs from a managed captive breeding program. These birds may act as a source for introduction of a novel disease agent to the wild population that may affect survival and reproduction. It is, therefore, essential to monitor and assess the health of OBPs and take appropriate measures to prevent and control the spread of viral infections. This requires knowledge of the existing virome to identify novel and emerging viruses and support development of appropriate measures to manage associated risk. By monitoring and protecting these animals from emerging viral diseases, we can help ensure their ongoing survival and preserve the biodiversity of our planet.

## INTRODUCTION

It is widely cited across literature that the presence of viral emerging infectious diseases (EIDs) that threaten avian species is increasing at an unprecedented rate, exacerbating biodiversity decline and species extinction (1 – 7). EIDs may arise in various ways, including mixing of different species in captivity, release of captive birds to the wild, introduction of exotic species to new environments, transmission between populations, or mutation of the viruses. Avian populations naturally harbor a range of viral pathogens, with healthy individuals possessing significant viral diversity and potentially acting as natural reservoirs of infection (8). Imbalances to the natural ecology of such populations may influence factors affecting exposure, transmission, and expression of disease caused by these viruses. Many viral outbreaks have been documented in avian populations over the past few decades, including high pathogenicity avian influenza (HPAI) (9 – 13), West Nile virus (6, 13 – 15), and numerous bacterial pathogens (6, 16 – 20), yet little is known about the natural virome of many captive Australian species, making it challenging to understand the risks around viruses and the impacts that they may have on such species.

A species of concern is the critically endangered, orange-bellied parrot (OBP) (*Neophema chrysogaster*), a small migratory ground parrot endemic to south-eastern Australia. With roughly 70 wild individuals and a captive insurance population of around 500 individuals, this species faces a high risk of extinction and remains heavily dependent on conservation interventions, such as the release of captive-bred birds to the wild (21). Various factors affect the conservation of this species, including a small wild population size, inbreeding depression, and associated decline in genetic diversity, which in turn increases the impacts of stochastic events and infectious disease outbreaks (21). Knowledge of infectious agents in this species is limited, with previous research having focused on viruses that have caused observed morbidity and/or mortality. One circovirus known to affect the species is beak and feather disease virus (BFDV), which has been shown to contribute to the death of wild and captive OBPs (22, 23); however, neither the prevalence of infection nor the ecological impacts of this virus on the captive and wild population are well understood (21). Similarly, psittacine siadenovirus F (PsSiAdV-F) has been found to be enzootic across the captive OBP population, although its impact on health and risk to the OBP population are unclear (2). Beyond this, our knowledge about the viruses of the OBP is limited; however, there is potential for the presence of novel viruses within these populations for which we have very little understanding of the impacts on health and reproduction and risks to conservation.

Before metagenomics, there was limited understanding of the viromes present in animal and human hosts. With the advent of metagenomics, information on eukaryotic and prokaryotic viruses and even on viruses that infect other viruses (virophages) has increased (24 – 29). Metagenomics or metatranscriptomics is a relatively new technique that enables the detection and characterization of entire microbiomes, including bacterial, viral, and host genomes, rather than singular species in isolation, in animals and human hosts (4, 5, 27). It employs either long-read platforms or short-read platforms, such as Illumina, a second-generational system which generates short reads in parallel, which are typically able to produce results with greater quantification, accuracy, and sensitivity (30, 31). These sequences are sifted via computational methods to produce detailed mathematical models without constraint of reference genome data (30, 31). These platforms sequence extracted genetic data from prepared libraries, and due to the decline in cost, metagenomic research has become more accessible (30). However, these techniques rely on reference catalogues, whether being entire genomes or sections of coding regions, yet many genomic reference databases possess considerable gaps in available references. Viral genomes are considerably smaller than other prokaryote genomes, with fewer genes encoded within. Viruses also possess greater diversity between taxa; thus, determining their role can prove difficult to characterize.

The aims of this study were to use metagenomics to detect and characterize known and novel viruses that may exist in the OBP captive breeding population, broadening our knowledge of viruses that may create risk to the future survival of this species.

## RESULTS

### Detected viruses within the captive orange-bellied parrot flocks

There were eight viral contigs detected in Aviary 1 (Table 1), with seven viral contigs identified from one sample (OBP1) and one viral contig identified from the second sample (OBP2). Of the seven viral contigs in OBP1, four were found to be complete genomes, with the remaining three being partial genome sequences, while the single contig from OBP2 represented the full genome of an adenovirus. These viral sequences showed high read counts, ranging from 710 to 29,436 reads, providing a great level of coverage across the entire sequence (average coverage ranged between 48.64× and 639.40×) (Table 1). The detected viruses belong to various families such as *Adenoviridae*, *Circoviridae*, and *Parvoviridae* (Table 1).

**TABLE 1:** Summary of the detected viruses in the Aviary 1

Aviary 1, orange-bellied parrot 1 (OBP1)								
Virus taxonomy	Virus	GenBank accession	Novel	COV^*a*^	Read count	Genome length, completeness	Best Blast hit (GenBank accession)	% Identity
Adenoviridae, Siadenovirus	Psittacine siadenovirus	OP377084	No	158.57	29,436	25,393 nt, yes	Psittacine siadenovirus F (MW365934)	99.96
Circoviridae	Curcivirus sp.	OP564891	Yes	272.68	7,829	3,935 nt, yes	DNA virus sp. (MZ244324)	97.43
Parvoviridae	Psittaciform parvoviridae sp.	OP577479	No	46.50	1,140	3,321 nt, no	Phoenicopteridae parvo-like hybrid virus (MW046375)	94.17
Parvoviridae	Psittaciform parvoviridae sp.	OP577481	Yes	74.94	1,508	2,738 nt, no	Accipiter gentilis parvoviridae sp. (MW046523)	99.81
Parvoviridae	Psittaciform parvoviridae sp.	OP577480	Yes	75.84	1,529	2,562 nt, no	Accipiter gentilis parvoviridae sp. (QTE03962)	37.85
CRESSDNA-viricota	CRESS virus sp.	OP564892	No	48.64	710	1,999 nt, yes	Wastewater CRESS DNA virus 1 (MK583726)	87.50
Circoviridae, unclassified	Tick-associated circular DNA virus	OP564893	Yes	639.40	9,661	1,850 nt, yes	Tick-associated circular DNA virus (ON668995)	100.0

Aviary 1, orange-bellied parrot 2 (OBP2)									
Virus taxonomy	Virus	GenBank accession		Novel	COV^*a*^	Read count	Genome length, completeness	Blast top hit (GenBank accession)	% Identity
Adenoviridae, Siadenovirus	Psittacine Siadenovirus	OP377085		No	926.43	172,651	25,383 nt, yes	Psittacine siadenovirus F (MW365934)	99.96

^*a*^ COV, average coverage/depth of unique reads per nucleotide.

Three viral contigs were detected in Aviary 2—all from one sample (OBP4). One contig represented a complete genome, and the remaining two were found to be partial genomes. These sequences had high read counts, ranging from 11,865 to 15,115, with a high average coverage ranged from 300× to 406×. The first two viral contigs detected belong to the taxonomic family of *Picornaviridae*, and the final virus belonged to the *Parvoviridae* family (Table 2).

**TABLE 2:** Summary of the detected viruses in Aviary 2

Virus taxonomy	Virus	GenBank accession	Novel	COV	Read count	Genome length, completeness	Blast top hit (GenBank accession)	% Identity
Riboviria; Picornavirus	Psittacine picornavirus 1	OP577483	Yes	300.43	15,115	6,549 nt, no	Beihai sipunculid worm virus 5 YP_009333461	29.77
Riboviria; Picornavirus	Psittacine picornavirus 2	OP577484	Yes	340.23	11,865	4,853 nt, no	Wuhan arthropod virus (YP_009342255)	26.46
Parvoviridae	Psittaciform ambidensovirus 1	OP577482	Yes	406.03	13,582	4.644 nt, yes	Parvoviridae sp. (BK022927)	71.88

### Detected virus described by taxonomic family Adenoviridae: evidence of ongoing infection of PsSiAdV-F in OBP

In this study, two complete genomes of psittacine siadenovirus F strains were sequenced from Aviary 1 (OBP1/FE1/02/2022 and OBP1/FE2/01/2022), with an average coverage of 158.57× and 926.43×, respectively. Genome analysis of the detected strains of PsSiAdV-F matched most closely with a known siadenovirus (SiAdV) genome, the PsSiAdV-F strain OBP2209 (percent identity >99.90 for both genomes), followed by the PsSiAdV-F strain S10/AU (percent identity, 99.84%) and PsSiAdV-F strain WVL19065-01E (OBP1, 99.76%; OBP2, 99.77%). These matches had a query coverage of approximately 99% (e-value, 0.0) for both OBP1 and OBP2, indicating a high similarity. Comparative analysis of two strains of PsSiAdV-F complete genomes (PsSiAdV-F; GenBank accession no. OP377084 and OP377085) sequences can be seen in Fig. S1 and Table S2. Both the strains of PsSiAdV-F sequenced in this study were predicted to contain 25 methionine-initiated open reading frames (ORFs) (numbered from left to right) that were annotated as putative genes, showing a high similarity with the PsSiAdV-F strain OBP2209 (GenBank accession no. MW365934) and PsSiAdV-C (GenBank accession no. MN687905), which were chosen for comparison (Fig. S1 and Table S2).

Comparative analysis of the protein sequences encoded by the predicted ORFs, using BLASTX and BLASTP, identified homologs with high sequence similarities for 23 of 25 ORFs (percent identity, ≥98.0%). The AA sequence similarities of the remaining two ORFs (ORF17 and ORF25) were significantly lower (34.4% and 66.3%, respectively) (Table S2). No unique genes were identified in the PsSiAdV-F genomes sequenced in this study. Of the 25 predicted ORFs, 20 were common/conserved siadenovirus gene products; the remaining five were hypothetical proteins. All the predicted protein-coding genes of PsSiAdV-F genomes sequenced in this study showed the highest sequence similarities with the genes of psittacine siadenovirus F strain OBP2209 at the range of 34% to 100% identity (Table S2).

**Fig 1:**
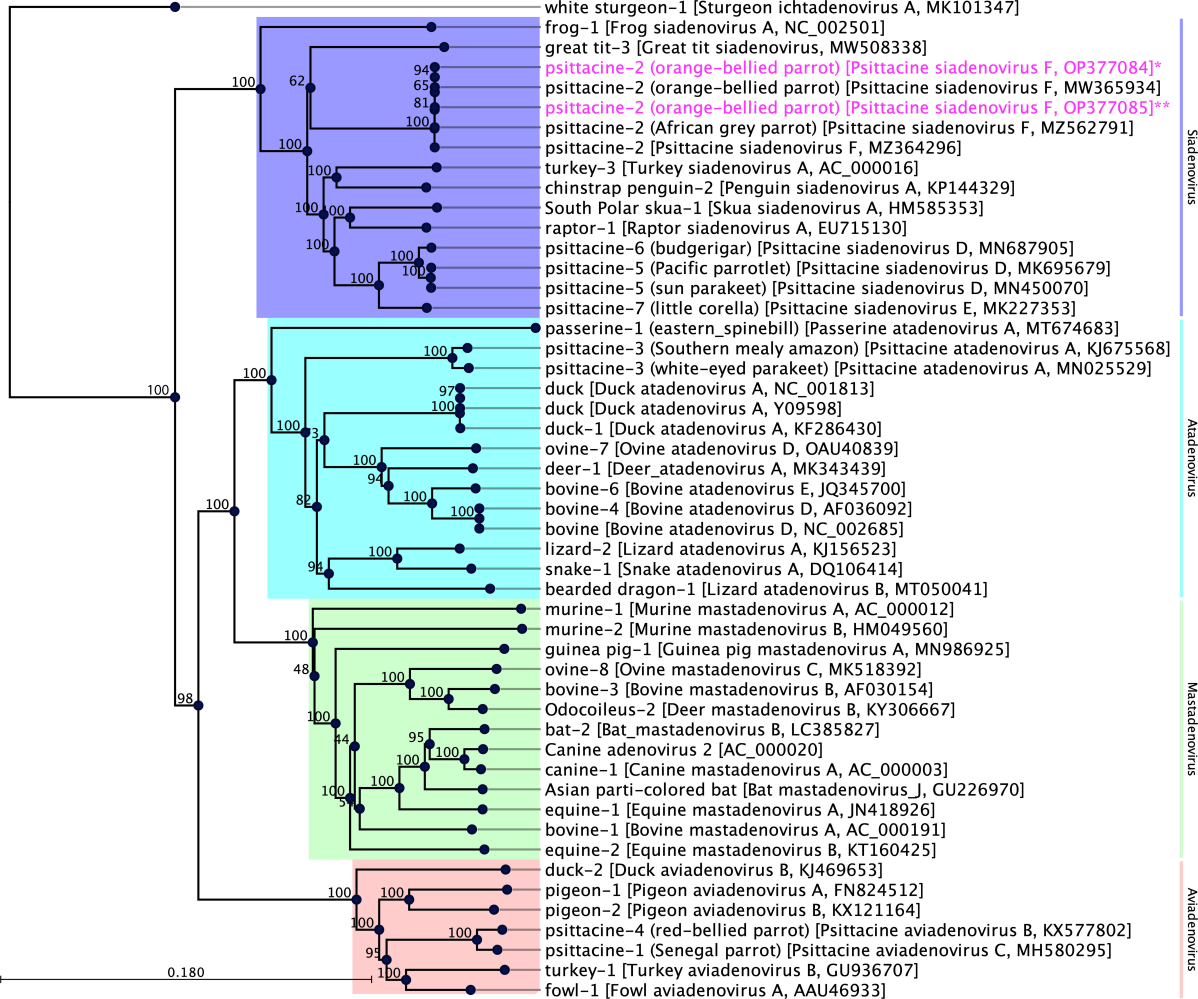
A maximum likelihood phylogenetic tree was generated, showing a possible evolutionary relationship of the PsSiAdV-F strain OBP/FE1/02/2022 and OBP/FE2/01/2022 (GenBank accession no. OP377084 and OP377085, respectively), with other selected adenoviruses. The phylogenetic tree was generated via a MAFFT alignment L-INS-I (version 7.450) of concatenated AA sequences of the complete DNA-dependent DNA polymerase protein (DNA pol), pTP, penton, and hexon genes utilizing Geneious Prime (version 2022.1.1, Biomatters, Ltd., Auckland, New Zealand). The scoring matrix BLOSUM62 and a gap open penalty of 1.53 were selected (offset value = 0.123). The tree was constructed with 1,000 bootstrap re-samplings in CLC Genomic Workbench (version 9.0.1). The numbers on the left show bootstrap values as percentages, and the labels at branch tips refer to original AdVs host species followed by AdVs name and GenBank accession numbers in brackets. The PsSiAdV-F strains detected within this study are highlighted in pink and asterisk.

Phylogenetic analysis was performed, based on concatenated AA sequences of two non-structural (DNA polymerase, pTP) and two structural (penton, hexon) protein sequences. The resulting maximum likelihood (ML) tree (Fig. 1) supported the inclusion of the two new sequences of the PsSiAdV-F strains (OBP1/FE1/02/2022 and OBP1/FE2/01/2022) in the *Siadenovirus* genus. The sequenced PsSiAdV-F strains OBP1/FE1/02/2022 and OBP1/FE2/01/2022 were positioned in a distinct subclade with PsSiAdV-F (GenBank accession no. MW365934), a sequence recovered from liver samples collected from a deceased captive OBP in 2020 (100% bootstrap support) (Fig. 1) (32).

### Numerous novel circoviruses and CRESS-DNA viruses

**Fig 2:**
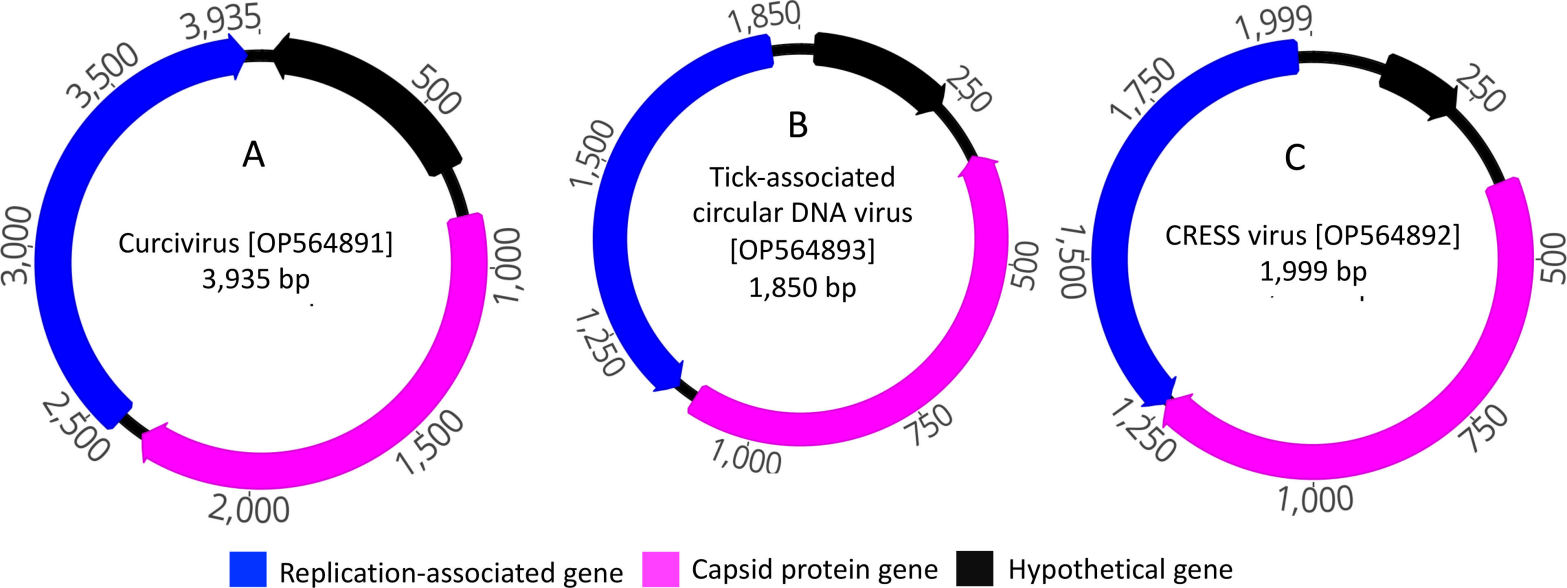
The colored arrows indicate the genes and ORFs encoded within the sequence, with their orientation representative of the direction of transcription. The ORFs are color coded following the conventions set in the legend. Within the three complete genomes, a rep-associated gene (blue), capsid-protein gene (pink), and a hypothetical protein (black) were identified. (**A**) Crucivirus, (**B**) tick-associated circular DNA virus, and (**C**) CRESS DNA virus.

Three unique circoviruses were detected in Aviary 1, including one crucivirus, a tick-associated DNA virus, and a CRESS-DNA virus. Genome analysis was conducted to identify genes, ORFs, or hypothetical genes, as illustrated in Fig. 2. There were no circoviruses identified from Aviary 2, and none of the detected circoviruses appeared to be BFDV.

The detected complete genome of a crucivirus strain OBP1/FE1/23/2022 in Aviary 1 was 3,935 bp in size (average coverage of 272.68×, GenBank accession no. OP564891). Genome analysis identified a match with a circovirus capsid-protein gene sequenced from a tick in China in 2021 (GenBank accession no. MZ244324), with an identity of 97.43% (query coverage, 14%; e-value, 0.0), followed by a crucivirus sequenced from a water sample in New Zealand (percent identity of 68.33%, GenBank accession no. MT263643). Further comparative analysis identified three ORFs (Fig. 2A) and found that the replication-associated gene and capsid-protein gene showed identities of 28.57% and 96.91% with the representative genes of a tick-associated DNA virus and crucivirus, respectively (Table S3). The detected complete genome of a tick-associated circular DNA virus strain OBP1/FE1/85/2022 within the OBP1 sample was 1,850 bp in size (average coverage of 639.40×, GenBank accession no. OPOP564893). Comparative analysis of the detected tick-associated circular DNA virus based on complete genome and their known functional protein coding genes showed a 100% identity with another tick-associated circular DNA virus sequenced from a tick (*Haemaphysalis japonica*) in China in 2022 (Fig. 2B and Table S3).

A CRESS DNA virus sp. strain OBP1/FE1/74/2022 was sequenced within the Aviary 1 (OBP1, GenBank accession no. OP564892). The complete genome of this virus had a length of 1,999 bp (average coverage of 48.64×) and found the most closely related known genome was a wastewater CRESS DNA virus (GenBank accession no. MK583726; identity of 87.50%), followed by an *Alphasatellitidae* isolate (GenBank accession no. MT203185.1; identity of 73.50%) and a *Circoviridae* isolate (GenBank accession no. MT203185.1, identity of 88.37%). However, these matches had a low query coverage. Additionally, the predicted ORFs also showed a low identities value (Fig. 2C and Table S3).

**Fig 3:**
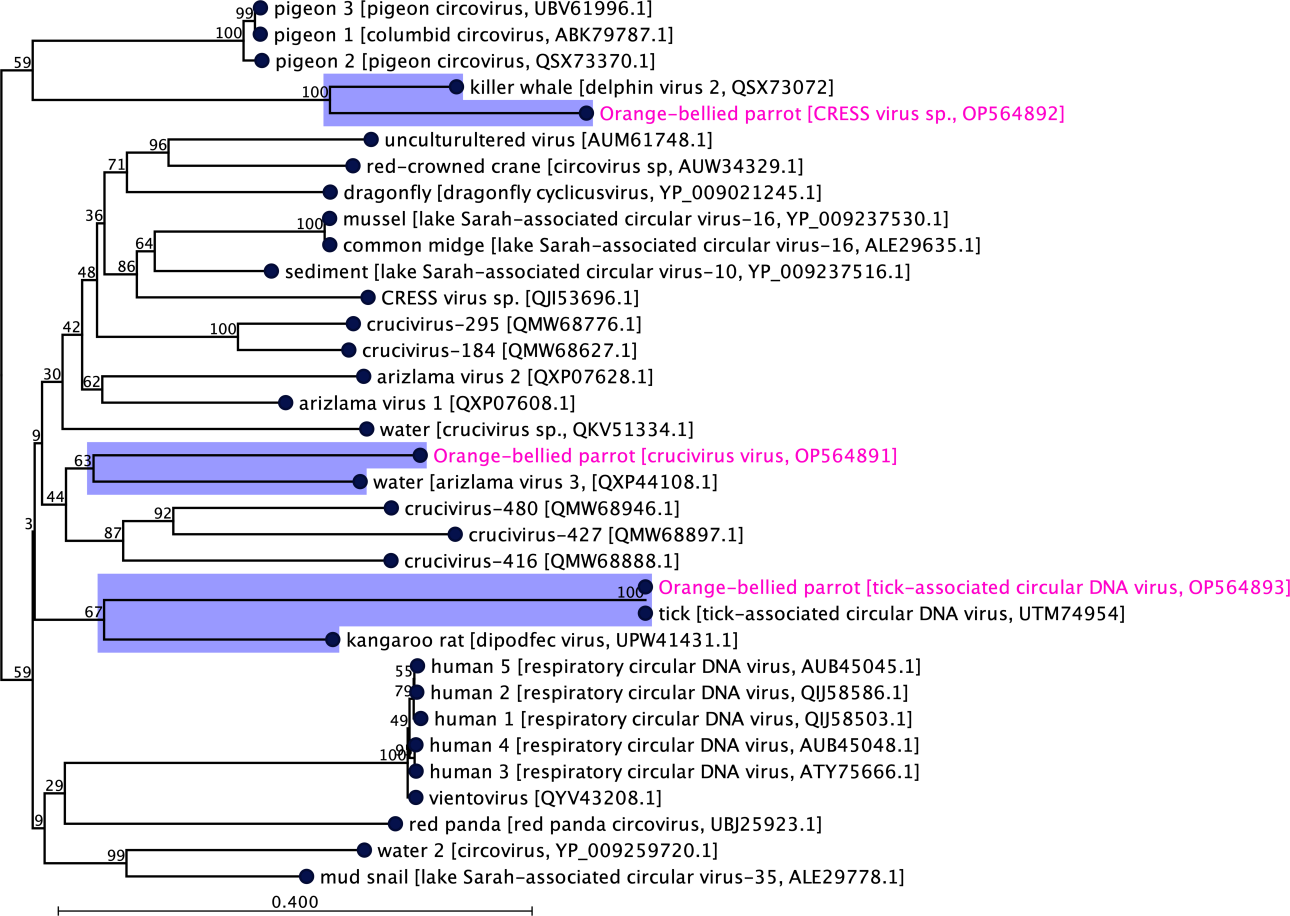
A maximum likelihood phylogenetic tree was generated, showing a possible evolutionary relationship of circular DNA viruses detected in this study (GenBank accession no. OP564891-93), with other selected circular DNA viruses. The tree was generated utilizing selected rep-associated protein coding gene sequences in CLC Genomic Workbench (version 9.0.1). The tree was constructed with 1,000 bootstrap re-samplings. The numbers on the left show bootstrap values as percentages, and the labels at branch tips refer to original host species (if host species are defined) followed by virus name and GenBank accession numbers in brackets. The circular DNA viruses detected within this study and related subclades are highlighted in pink color and blue background, respectively.

In the resulting ML based on conserved rep-associated protein coding gene of the selected circular DNA viruses (Fig. 3), the crucivirus (GenBank accession no. OP564891) detected in this study showed the closest evolutionary link with arizlama virus 3 (GenBank accession no. QXP44108.1) sourced from an environmental sample in France. This may indicate that crucivirus detected in this OBP population may not have a close relative of Australian origin or has not yet been described. The tick-associated circular DNA virus sequenced in this study (GenBank accession no. OP564893) emerged as a novel subclade with two other circular DNA viruses, one sequenced from tick (*Haemaphysalis japonica*, GenBank accession no. UTM74954) in China and another one from kangaroo rat (*Dipodomys merriami*, GenBank accession no. UPW41431) in the United States of America (USA) indicating that the circular DNA virus found in this study may have originated from a likely common progenitor. Finally, the CRESS virus discovered in this study (GenBank accession no. OP564892) appears to be most closely evolutionary related to the delphin virus 2 found in kidney tissue of killer whale (*Orcinus orca*, GenBank accession no. QSX73072) in the USA in 2021 and positioned in a novel subclade (Fig. 3).

### Parvoviridae: evidence of novel parvoviruses in the OBP population

Within Aviary 1, three parvoviruses were detected, two of which matched most closely with viruses isolated from a Northern Goshawk (*Accipiter gentilis*) (Table 1). The three parvoviruses had identities between 37.85% and 99.81%, but most of them had a low query coverage (Table 1). At gene level, Psittaciform parvoviridae sp. strain OBP1/FE1/44/2022 (GenBank accession no. OP577481) showed a significant identity with the representative parvoviruses (replication-associated gene, NS1, and capsid gene, VP1, 66.77% and 39.77%, respectively) (Table S4), whereas another parvovirus, Psittaciform parvoviridae sp. strain OBP1/FE1/49/2022 (GenBank accession no. OP577480), showed a low identity at NS1 gene level (28.82%) compared to the parvovirus sequenced from Northern goshawk (*Accipiter gentilis*) in China (GenBank accession no. QTE03962) (Table S4). The third parvovirus, Psittaciform parvoviridae sp. strain OBP1/FE1/29/2022 (GenBank accession no. OP577479), showed the highest identities (94.17%) with a parvo-like hybrid virus sequenced from anal swabs of Psittacidae bird in China (GenBank accession no. MW046375).

The complete genome of a psittaciform ambidensovirus 1 strain OBP4/DE2/13/2022 was identified from Aviary 2. The viral contig was 4,644 bp in size (GenBank accession no. OP577482) and surrounded by two matching inverted terminal repeat regions, constituting 264 bp each. The genome showed the highest identity (71.88%) with another Ambidensovirus sequenced from a human metagenomic study in the USA (GenBank accession no. BK022927.1) (33); however, there was a very low query coverage (5%). Consequently, three predicted ORFs also showed low identities with the representative gene sequences of parvoviruses, ranging from 25.77% to 43.13% (Table S4).

**Fig 4:**
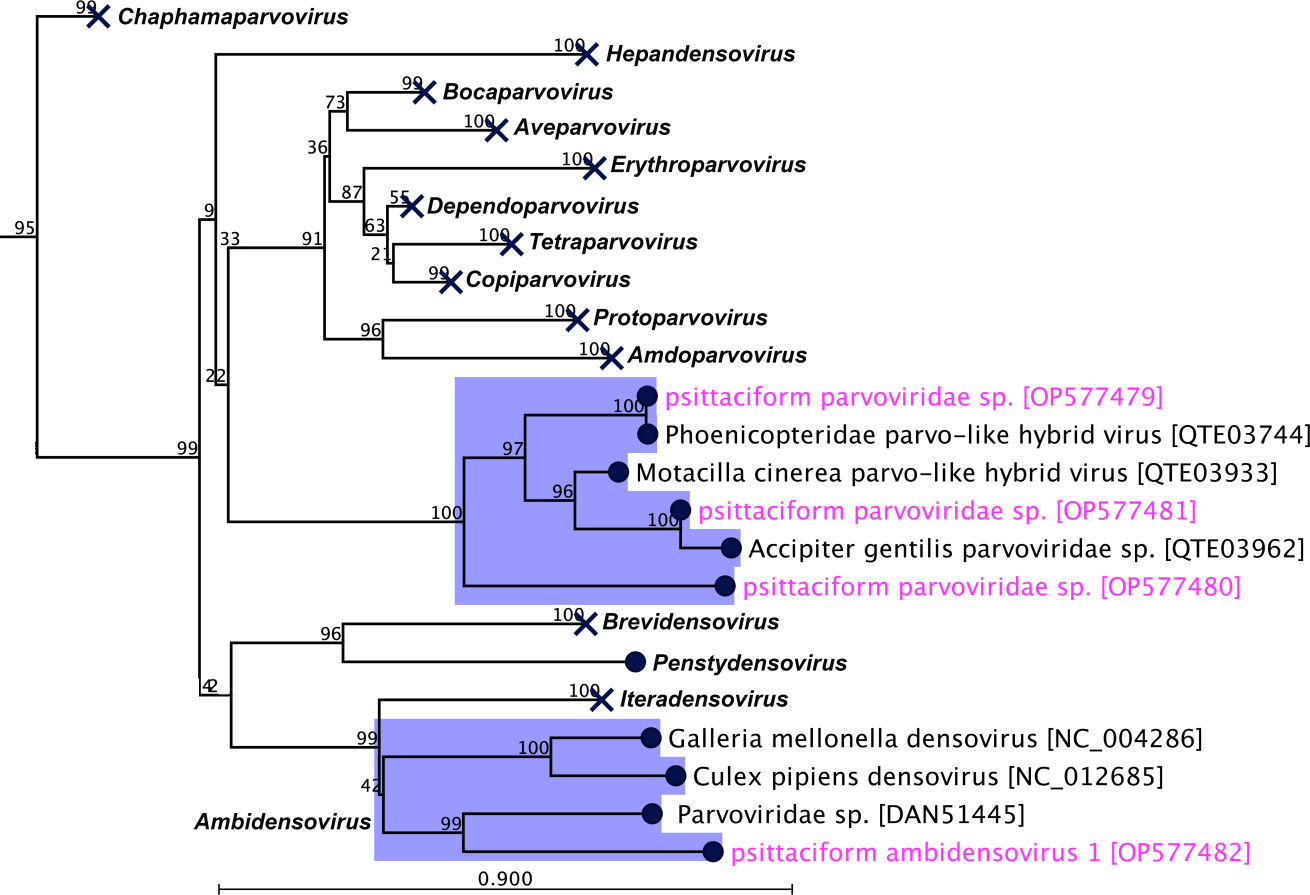
A maximum likelihood phylogenetic tree was generated, showing a possible evolutionary relationship of parvoviruses detected in this study (GenBank accession no. OP564879-82), with other selected parvoviruses. The tree was generated utilizing selected rep-associated protein coding gene (NS1) sequences in CLC Genomic Workbench (version 9.0.1). The tree was constructed with 1,000 bootstrap re-samplings. The numbers on the left show bootstrap values as percentages, and the labels at branch tips refer to virus name followed by GenBank accession numbers in brackets. The parvoviruses detected within this study and related subclades are highlighted in pink color and blue background, respectively. Except two clades related to this study, all other clades are collapsed (please see Supplementary tree S2 for details).

In the resulting ML tree (Fig. 4) using the conserved rep-associated NS1 protein coding sequences, the three parvoviruses (GenBank accession no. OP577479-81) sequenced from the Aviary 1 (highlighted in pink) in this study emerged as novel lineages with other three *Parvoviridae* sp., indicating that these viruses may be circulating within the population and have not yet been reported. The psittaciform ambidensovirus 1 (GenBank accession no. OP577482) detected Aviary 2 in this study showed the closest evolutionary link with a Parvoviridae sp. (GenBank accession no. DAN51445) sequenced from a human metagenomic study in the USA (33) and two other ambidensoviruses (Fig. 4).

### Picornaviridae

In this study, a partial genome of psittacine picornavirus 1 strain OBP4/DE2/3/2022 (6,549 bp in length, average coverage of 300.43×, GenBank accession no. OP577483) was detected in the Aviary 2 (sample OBP4). Nonetheless, BlastN analysis failed to identify a match with any viruses; predicted ORFs showed a good match (29.77% identity) with the hypothetical protein 1 gene of Beihai sipunculid worm virus 5 sequenced from peanut worms in China (Table S5) (27). Another picornavirus detected within Aviary 2 was a partial genome (4,853 bp in size, average coverage 340.23×, GenBank accession no. OP577484) of psittacine picornavirus 2 strain OBP4/DE2/9/2022, which showed the closest match (26.46% identity) with Wuhan arthropod virus (GenBank accession no. YP_009342255). Further comparative analysis on the predicted ORFs showed that the two of three ORFs were linked with picornaviruses of arthropod origin (Table S5).

**Fig 5:**
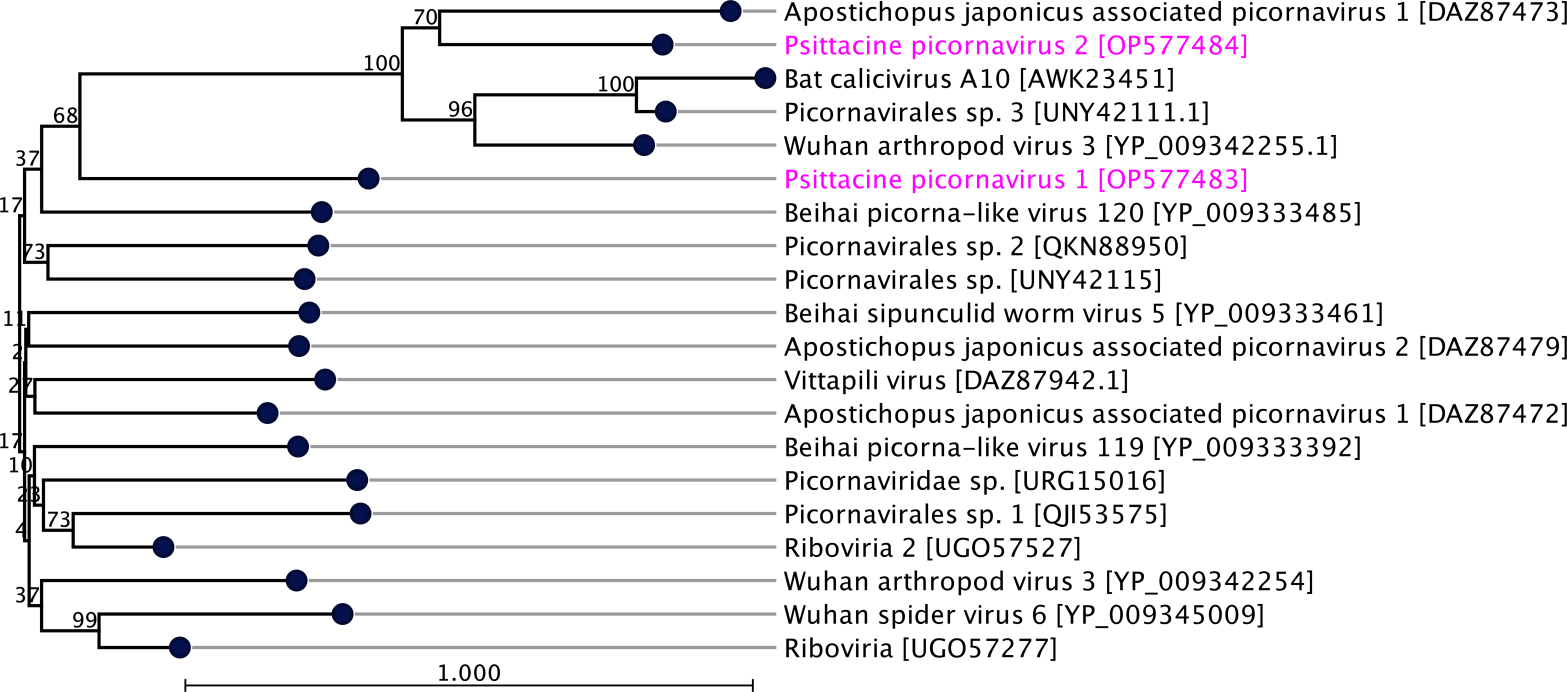
A maximum likelihood phylogenetic tree was generated, showing a possible evolutionary relationship of picornaviruses detected in this study (GenBank accession no. OP577483-84), with other selected picornaviruses. The tree was generated utilizing selected rep-associated protein coding gene (NS1) sequences in CLC Genomic Workbench (version 9.0.1). The tree was constructed with 1,000 bootstrap re-samplings. The numbers on the left show bootstrap values as percentages, and the labels at branch tips refer to virus name followed by GenBank accession numbers in brackets. The picornaviruses detected within this study and are highlighted in pink color.

Phylogenetic analysis based on the single hypothetical protein sequences or structural RNA-dependent polymerase gene sequences of the selected picornaviruses supported the inclusion of the two new psittacine picornavirus sequences (GenBank accession nos. OP577483 and OP577484, respectively) in the *Picornaviridae* family (Fig. 5). The two picornaviruses sequenced within this study do not appear to be closely related, having diverged quite early from each other. The first picornavirus sequenced within this study is a psittacine picornavirus 1 (GenBank accession no. OP577483) emerged in a unique subclade; however, it showed a likely evolutionary link with another picornavirus, Beihai sipunculid worm virus 5 sequenced from China (GenBank accession no. YP_009333461) (27). The second picornavirus isolated in this study is a psittacine picornavirus 2 (GenBank accession no. OP577484) that emerged in a subclade with four other picornaviruses. The psittacine picornavirus 2 appears to be most closely related to a virus isolated from a sea cucumber (*Apostichopus japonicus)* (GenBank accession no. DAZ87473 DAZ87473) in the USA in 2022 (Fig. 5).

## DISCUSSION

Metagenomics is a widely adopted technique for screening and characterizing microbes including viruses and bacteria and can provide more insight into the diversity of such microbiomes in poorly studies populations (30, 31, 34), such as the orange-bellied parrot (*Neophema chrysogaster*). Furthermore, metagenomic studies on these populations can facilitate the development of preventative and mitigation strategies to preserve endangered species (1). Many recent viral outbreaks involve avian hosts, such as the H5N1 avian influenza (12). However, there remains a considerable gap in the literature regarding the evolution and spread of viruses within captive populations of endangered Australian parrots, including the presence of any potentially unidentified strains or their contribution to the decline of the endangered species (1). The OBP remains at risk of the impacts from the emergence of a novel disease; however, their existing virome is presently uncharacterized. This study aimed to explore this gap and characterize the virome of a subpopulation of captive OBPs via a high-throughput next-generation sequencing technology to determine viral diversity.

Eleven viruses were successfully detected and characterized from the extracted nucleic acids, coming from a range of taxa, including two within the *Siadenovirus* genus, three unique circoviruses, four parvoviruses, and two picornaviruses. Interestingly, there was greater viral diversity found in samples from Aviary 1, with 8 of 11 viruses exclusive to this aviary, and only one virus identified that was common to both aviaries: PsSiAdV-F strain. The first two sequences detected in Aviary 1 were complete genomes of PsSiAdV-F strain, an expected finding given previous detections within captive OBP population (32). The three circoviruses were sequenced from OBPs in Aviary 1, including a crucivirus and circovirus, both of which appeared to most closely resemble a tick-associated virus isolated in China (GenBank accession nos. MZ244324 and MT263643, respectively), as well as a CRESS DNA virus, which matched most closely with a virus isolated from wastewater in Florida, USA (GenBank accession no. MK583726). In addition to these viruses, interestingly, a total of four parvoviruses were detected within this study (GenBank accession no. OP577479-81) with three being isolated from Aviary 1.

The *Adenoviridae* family is made up of six recognized genera (*Atadenovirus*, *Aviadenovirus*, *Ichtadenovirus*, *Mastadenovirus*, *Siadenovirus*, and *Testadenoviru*s) and contain a linear, medium-sized, non-enveloped dsDNA genome. Many adenoviruses are pathogenic, typically occupying the respiratory and gastrointestinal tracts in various vertebrate hosts (humans, birds, and ungulates) (35). Among them, PsSiAdV-F under the genus *Siadenovirus* had been reported previously in the OBP, elegant parrot (*Neophema elegans*), scarlet-chested parrot (*Neophema splendida*), and an African gray parrot (2, 32, 36). Avian SiAdVs are prevalent in both wild and captive avian populations, having been detected across all continents (3, 37). The resulting ML tree of adenoviruses (Fig. 1) showed that the siadenoviruses detected in this study were placed in a monophyletic clade alongside the PsSiAdV-F sequenced from the liver sample of a deceased captive OBP in 2020. Considering the phylogenetic position and genome-wide identities (>99%) among PsSiAdV-F sequenced so far, this study provides further evidence that ongoing infection or reinfection is occurring within the captive OBP. The release of captive-bred OBP harbors the risk of introducing PsSiAdV-F into the wild population, which may affect their survival and reproduction. Thus, it is critical that the health of captive OBPs is monitored, the spread of viral agents is controlled, and the conservation of the species is ensured. However, it is difficult to confer the biological consequences of this PsSiAdV-F from this study.

Members of the *Circoviridae* family are small, circular ssDNA viruses that range from 1.7 kb to 2.1 kb in size, with two major ORFs encoding a rep-associated protein gene and a capsid-protein gene (38), and CRESS viruses are small, rep-associated, circular ssDNA. They can be found in various families, including *Alphasatellitidae*, *Genomoviridae*, and *Circoviridae* (38). Circoviruses possess one of the highest mutation rates among DNA viruses (39, 40), which significantly contribute to the adaptability of these viruses in changing environments. It is evident that circoviruses, such as BFDV, are persistently detected in psittacine birds, which is particularly concerning for a critically endangered species, given its pathogenesis and high mortality rate (2, 22, 23, 41, 42). A prior study conducted by Sarker (2) had identified BFDV along with other circoviruses sampled from two other species within the same genus (*Neophema*) as OBP, the elegant parrot (*Neophema elegans*), and scarlet-chested parrot (*Neophema splendida*). So, the detection of some circoviruses during this study is not surprising. Notably, no BFDV was detected from the samples analyzed in this study, indicating no current infection with BFDV. However, whether previous infections had occurred cannot be determined. Subsequent phylogenetic tree analysis using the conserved rep-associated genes positioned the detected circoviruses in distinct clades (Fig. 3), highlighting an unknown evolutionary history.

The *Parvoviridae* family, made up of the *Parvovirinae* and *Densovirinae* subfamilies, encompasses non-enveloped ssDNA viruses, typically 4–6 kb in length and 25 nm in diameter (43). The *Parvovirinae* viruses are often isometrically shaped, infecting the small intestine and bone marrow of vertebrate host cells of mammals and birds (43). Recently, many novel parvoviruses were detected across a range of hosts, from pigs, rats, and a range of avian species (44), yet little is known about the pathology of avian parvoviruses or their transmission between hosts, particularly within the *Neophema* genus. In a study conducted by Sarker (2), parvovirus genomes were identified and characterized in two other species of *Neophema* parrots. As OBPs belong to the same genus, this study sought to determine what novel parvoviruses were present within the captive OBP insurance population. A phylogenetic tree was produced (Fig. 4) comparing the evolutionary relationship between the four parvoviruses detected, which had indicated a strong phylogenetic signal between the three viruses detected in Aviary 1, and a strong bootstrap support (100%), as well as positioning them in a monophyletic clade. Interestingly, the fourth parvovirus psittaciform ambidensovirus 1 strain shares a close phylogenetic relationship with several densoviruses and has been placed in a clade alongside some viruses that belong to the genus *Ambidensoviru*s. The evolution of the virus and its route of transmission are unknown, warranting further investigation.

Non-avian host-associated picornaviruses have regularly been detected within wild and captive Australian birds (4, 5). Viruses under the family *Picornaviridae* are non-enveloped and contain a positive-sense, single-stranded RNA genome that often transcribes RNA-dependent RNA polymerase in the reverse direction (44). Picornaviruses infect many vertebrates, including mammals, birds, and wild animals (2, 4). These insect-host-associated viruses are commonly detected within avian hosts. This suggests that the transmission of non-avian viruses into avian hosts is potentially via contaminated feed; direct consumption during transportation (2); parasites, such as lice and mites; or vectors, such as mosquitos and biting flies. Sarker (2) had also characterized a picorna-like virus, which showed a close evolutionary link with picorna-like viruses sequenced from flying insects. In this study, the detected viruses appear to be unique, given the lack of comparable NCBI BlastN and BlastP matches, and seem to share some common ancestors with other insect-hosted picornaviruses, as indicated by Fig. 5. This suggests that the virome of the captive OBP population encompasses a diverse virome that is not well characterized or understood, in addition to a significant gap in genomic data available for such viruses. Further study is necessary in sequencing more circoviruses to enable a clearer understanding of the phylogenetic relationship between avian circoviruses, particularly non-BFDV strains. Moreover, the impact of a limited database is further highlighted by the genomic analysis results, which found that the closest match to the detected crucivirus and tick-associated virus had a low query coverage of 14% and 12%, respectively, despite the high percentage identities (97.43% and 100%, respectively).

There are some limitations to the genomic and phylogenetic analysis techniques utilized in this study, including a notable gap in the database of sequenced viruses available for comparison. As indicated by the ML phylogenetic tree produced from the circoviruses detected within this study (Fig. 3), the lack of sequence data available can result in poor or varying confidence scores in the analysis of evolutionary relationships, such as query coverage and ML bootstrap value, and determining the significance of such finding may prove challenging. Furthermore, neither previous viral infections can be identified, nor future infection impacts can be predicted from these findings; thus, regular diagnostics would be necessary to identify the evolution and transmission of novel, unique, or significant pathogens. Expanding the database of sequenced viruses is essential for accurate evolutionary analysis, including the route of transmission and presence within populations.

This study has identified existing and novel viruses of *Adenoviridae*, *Circoviridae*, *Parvoviridae*, and *Picornaviridae* within this captive OBP population of which many appear to be unique and unreported. Critically, the findings of this study reflect only the captive population at one facility and does not reflect that of the wild OBP population or across other OBP captive insurance populations, highlighting the necessary continuation of research on the species virome, both across the captive and wild populations, to provide much needed clarification on the pathogen-host relationship, the evolutionary relationships, and likely transmission routes between populations. As many conservation methods require a comprehensive understanding of the species’ virome, the characterization of wild OBP viromes could provide insight into the predominant viruses threatening the species. Furthermore, determining the pathogen-host interactions and how they interact with other drivers of extinction may aid the development of a more appropriate conservation management strategy. Determining what viruses are present within the wild populations would enable comparison and identification of potential emerging viruses and risks that may be introduced from captive-bred OBPs released to the wild as part of conservation support strategies to support the wild population.

## MATERIALS AND METHODS

### Sampling and ethical consideration

Fresh fecal samples were collected from captive OBPs maintained as part of the captive insurance population in Werribee Open Range Zoo, Victoria. Two pooled fecal samples were collected from each of two aviaries at the same institution (Aviary 1 = OBP1 and OBP2; Aviary 2 = OBP 3 and OBP4), each of which housed between 10 and 14 birds at the time of sampling. Fecal specimens were obtained from the food trays and aviary flooring during provision of routine animal care and were stored at −80°C in a cryovial with RNALater until further processing. Sample collection did not require handling or manipulation of OBPs, although it was conducted under approval from the Zoos Victoria Animal Ethics Committee (ZV21005). The Animal Ethics Committee at La Trobe University was informed that findings from the material (with no bird touching) were to be used in a publication, and a formal waiver of ethics approval was granted.

### Virus enrichment and virus nucleic acid extraction

Potential impurities, such as host cells, bacteria, food particles, and free nucleic acids, from the fecal samples were removed, which was followed by enrichment of virus particles performed as per stated methods (5), with mirror variations. Briefly, the fecal material was aseptically resuspended and homogenized vigorously in sterile phosphate-buffered saline (PBS) (1:10) and centrifuged at 17,000 × *g* for 3 minutes at room temperature. The supernatant was filtered using a 0.80-µm syringe filter, and the filtrate was processed downstream. The samples were then ultracentrifuged at 178,000 × *g* for 1 hour (30 psi for 1 hour) at 4°C using the Hitachi Ultracentrifuge CP100NX. The supernatant was discarded, and the pellet was suspended in 130 µL of sterile PBS. The filtrates were then nuclease treated using 2 µL of benzonase nuclease (25–29 U/µL, purity  >90%, Millipore) and 1 µL of micrococcal nuclease (2,000,000 gel units/mL, New England Biolabs) and incubated at 37°C for 2 hours. The nuclease reaction was stopped by adding 3 µL of 500 mM ethylenediaminetetraacetic acid. Viral nucleic acids were extracted using the QIAamp Viral RNA Mini Kit (Qiagen, Valencia, CA, USA), without adding any carrier RNA, which allowed the extraction of both viral DNA and RNA simultaneously. The quantity and quality of the isolated nucleic acids were determined using Nanodrop and an Agilent Tape Station (Agilent Technologies, Mulgrave, VIC, Australia) by the Genomic Platform, La Trobe University.

### Next-generation sequencing

Before library construction, extracted nucleic acids were subjected to cDNA synthesis, and amplification was carried out using the Whole Transcriptome Amplification Kit (WTA2, Sigma-Aldrich, Darmstadt, Germany) as per manufacturer’s instructions. Amplified PCR products were then purified using the Wizard SV Gel and PCR Clean-Up kit (Promega, Madison, WI, USA). The quantity and quality of the purified product were checked using a Qubit dsDNA high sensitivity assay kit with Qubit Fluorometer v3.0 (Thermo Fisher Scientific, Waltham, MA, USA).

The library construction was performed on the four pooled samples using the Illumina DNA Prep (Illumina, San Diego, CA, USA) as per kit instructions, starting with 250 ng of DNA as measured by Qubit (Invitrogen). The quality and quantity of the prepared libraries were assessed by the Australian Genome Research Facility, Melbourne, Australia. The prepared libraries were normalized and pooled in equimolar quantities. The quality and quantity of the final pooled libraries were further assessed as described above before sequencing by the facility. According to the manufacturer’s instructions, cluster generation and sequencing of the libraries were performed with read lengths of 150 bp paired-end on Illumina NovaSeq chemistry.

### Bioinformatic analyses

Preliminary quality evaluation for all raw reads was generated and pre-processed to remove ambiguous base calls and poor-quality reads and then trimmed to remove the Illumina adapter sequences. More than 37.99 million reads per library were generated, of which 35.59 million reads per library (93.21%) remained after adapter sequence and poor-quality read trimming, where 10 nucleotides were trimmed from both 5′ ends (Table S1). Trimmed sequence reads were mapped against the chicken genome (*Gallus gallus*, GenBank accession no. NC_006088) to remove likely host DNA contamination. In addition, reads were further mapped to *Escherichia coli* bacterial genomic sequence (GenBank accession no. U00096) to remove possible bacterial contamination, which yielded an average of 28.06 million unmapped reads (78.99%) per library (Table S1), which were used for *de novo* viral assembly. Unmapped reads were used as input data for *de novo* assembly using a SPAdes assembler (version 3.10.1) (45) under the “careful” parameter in the LIMS-HPC system (a high-performance computer specialized for genomics research in La Trobe University). The resulting contigs were compared against the nonredundant nucleotide and protein databases on GenBank using BLASTN and BLASTX (46), respectively, with an E-value threshold of 1 × 10^−5^ to remove potential false positives. Contigs that were significant BLAST hits with bacteria, eukaryotes, or fungi were filtered out to remove non-viral reads. Non-phageous viral contigs of interest greater than 300 nucleotides were imported in Geneious Prime software (Biomatters Ltd., New Zealand, version 2022.1.1) for further functional analysis. Average coverage of the viral contigs was calculated using the clean raw reads in CLC Genomics Workbench (version 9.0.1).

### Functional annotations

The assembled complete and partial viral genomes detected in this study were annotated as per stated protocol (2, 47) using Geneious Prime (version 2022.1.1, Biomatters, Ltd., Auckland, New Zealand). The viral taxonomy was determined through comparative analysis via GenBank’s BlastN, BlastX, and BlastP, with the highest match being chosen against selection criteria (E-value ≤0.0, percentage identity >35%). Annotation of the ORFs in the identities of viral genomes was completed by comparison against specific criteria (>30 AA, methionine start codon, <50% overlap of a gene) via NCBI’s database. The genome for all the detected circoviruses was annotated using Geneious software (version 2022.1.1, Biomatters, New Zealand), where representative circovirus genomes were used as reference guidelines (2, 48). The identified ORFs were converted into FASTA files and compared for similarity using both NCBI’s BlastP and BlastX. They were then compared against conserved domain databases (NCBI, Bethesda, MA, USA) (46). This was done to determine whether any of the ORFs or proteins shared a highly similar sequence.

### Comparative genomic and phylogenetic analyses

Genomic comparison of the newly sequenced complete viral genomes was visualized using Geneious (version 2022.1.1). The sequence similarity between the selected viral sequences was identified against representative viral sequences by MAFFT alignment L-INS-I in Geneious (version 2022.1.1, Biomatters, Ltd., Auckland, New Zealand).

For phylogenetic analyses, representative viral genome or gene sequences were downloaded from GenBank, and virus-specific trees were constructed using CLC Genomics Workbench (version 9.0.1) and Geneious software (version 2022.1.1, Biomatters, New Zealand). Amino acid sequences of protein-coding genes and nucleotide sequences of the selected partial genes were aligned using the MAFTT L-INS-I algorithm implemented in Geneious (version 7.388) (49). To determine the best-fit model to construct phylogenetic analyses, a model test was performed using CLC Genomics Workbench (version 9.5.4) using default parameters, favoring a general-time-reversible model gamma distribution rate variation and a proportion of invariable sites (GTR + G + I). Phylogenetic analyses for nucleotide and protein sequences were performed using the GTR and WAG substitution model, respectively, with 1,000 bootstrap support in CLC Genomics Workbench (version 9.0.1).

## Data Availability

The study sequences are available in the National Centre for Biotechnology Information (NCBI) under BioProject accession number PRJNA984348 (BioSample accessions: SAMN35766466-SAMN35766469). The raw Illumina sequence read data generated in this study have been deposited to the NCBI sequence read archive [SRA (https://www.ncbi.nlm.nih.gov/sra)] under the accession number SRR24962688-SRR24962691. The assembly viral sequences have been deposited to GenBank under the accession numbers MZ364296, MZ364298-MZ364305, MZ561459-MZ561460, and MZ645220. The software used to analyze raw sequence reads is described in Materials and Methods. The authors confirm that all supporting data have been provided in the article or supplementary data files.

## SUPPLEMENTAL MATERIAL

Supplemental File spectrum.03052-23-s0001.pdf Fig. S1-S2; Tables S1-S5.

ASM does not own the copyrights to Supplemental Material that may be linked to, or accessed through, an article. The authors have granted ASM a non-exclusive, world-wide license to publish the Supplemental Material files. Please contact the corresponding author directly for reuse.

## References

[1] Smith KF , Acevedo‐Whitehouse K , Pedersen AB . 2009. The role of infectious diseases in biological conservation. Animal Conservation 12:1–12. doi:10.1111/j.1469-1795.2008.00228.x

[2] Sarker S . 2021. Metagenomic detection and characterisation of multiple viruses in apparently healthy Australian neophema birds. Sci Rep 11:20915. doi:10.1038/s41598-021-00440-1 34686748 PMC8536680

[3] Vaz FF , Raso TF , Agius JE , Hunt T , Leishman A , Eden J-S , Phalen DN . 2020. Opportunistic sampling of wild native and invasive birds reveals a rich diversity of adenoviruses in Australia. Virus Evol 6:veaa024. doi:10.1093/ve/veaa024 32411389 PMC7211397

[4] Vibin J , Chamings A , Klaassen M , Alexandersen S . 2020. Metagenomic characterisation of additional and novel avian viruses from Australian wild ducks. Sci Rep 10:22284. doi:10.1038/s41598-020-79413-9 33335272 PMC7747739

[5] Vibin J , Chamings A , Collier F , Klaassen M , Nelson TM , Alexandersen S . 2018. Metagenomics detection and characterisation of viruses in faecal samples from Australian wild birds. Sci Rep 8:8686. doi:10.1038/s41598-018-26851-1 29875375 PMC5989203

[6] Staley M , Bonneaud C . 2015. Immune responses of wild birds to emerging infectious diseases. Parasite Immunol 37:242–254. doi:10.1111/pim.12191 25847450

[7] Patz JA , Daszak P , Tabor GM , Aguirre AA , Pearl M , Epstein J , Wolfe ND , Kilpatrick AM , Foufopoulos J , Molyneux D , Bradley DJ . 2004. Unhealthy landscapes: policy recommendations on land use change and infectious disease emergence. Environ Health Perspect 112:1092–1098. doi:10.1289/ehp.6877 15238283 PMC1247383

[8] McCallum H . 2012. Disease and the dynamics of extinction. Philos Trans R Soc Lond B Biol Sci 367:2828–2839. doi:10.1098/rstb.2012.0224 22966138 PMC3427566

[9] Olsen B , Munster VJ , Wallensten A , Waldenström J , Osterhaus ADME , Fouchier RAM . 2006. Global patterns of influenza a virus in wild birds. Science 312:384–388. doi:10.1126/science.1122438 16627734

[10] Ellis TM , Bousfield RB , Bissett LA , Dyrting KC , Luk GSM , Tsim ST , Sturm-Ramirez K , Webster RG , Guan Y , Malik Peiris JS . 2004. Investigation of outbreaks of highly pathogenic H5N1 avian influenza in waterfowl and wild birds in Hong Kong in late 2002. Avian Pathol 33:492–505. doi:10.1080/03079450400003601 15545029

[11] Sharma G , Champalal Sharma D , Hwei Fen L , Pathak M , Bethur N , Pendharkar V , Peiris M , Altmeyer R . 2013. Reduction of influenza virus-induced lung inflammation and mortality in animals treated with a phosophodisestrase-4 inhibitor and a selective serotonin reuptake inhibitor. Emerg Microbes Infect 2:e54. doi:10.1038/emi.2013.52 26038487 PMC3821288

[12] Feare CJ . 2010. Role of wild birds in the spread of highly pathogenic avian influenza virus H5N1 and implications for global surveillance. Avian Dis 54:201–212. doi:10.1637/8766-033109-ResNote.1 20521633

[13] Brown JD , Stallknecht DE , Swayne DE . 2008. Experimental infection of swans and geese with highly pathogenic avian influenza virus (H5N1) of Asian lineage. Emerg Infect Dis 14:136–142. doi:10.3201/eid1401.070740 18258093 PMC2600149

[14] Hayes EB , Komar N , Nasci RS , Montgomery SP , O’Leary DR , Campbell GL . 2005. Epidemiology and transmission dynamics of West Nile virus disease. Emerg Infect Dis 11:1167–1173. doi:10.3201/eid1108.050289a 16102302 PMC3320478

[15] Marra PP , Griffing SM , McLean RG . 2003. West Nile virus and wildlife health. Emerg Infect Dis 9:898–899. doi:10.3201/eid0907.030277 12899147 PMC3023427

[16] Nolan PM , Hill GE , Stoehr AM . 1998. Sex, size, and plumage redness predict house finch survival in an epidemic. Proc R Soc Lond B 265:961–965. doi:10.1098/rspb.1998.0384

[17] Lawson B , Robinson RA , Colvile KM , Peck KM , Chantrey J , Pennycott TW , Simpson VR , Toms MP , Cunningham AA . 2012. The emergence and spread of finch trichomonosis in the British isles. Philos Trans R Soc Lond B Biol Sci 367:2852–2863. doi:10.1098/rstb.2012.0130 22966140 PMC3427565

[18] Robinson RA , Lawson B , Toms MP , Peck KM , Kirkwood JK , Chantrey J , Clatworthy IR , Evans AD , Hughes LA , Hutchinson OC , John SK , Pennycott TW , Perkins MW , Rowley PS , Simpson VR , Tyler KM , Cunningham AA . 2010. Emerging infectious disease leads to rapid population declines of common British birds. PLoS One 5:e12215. doi:10.1371/journal.pone.0012215 20805869 PMC2923595

[19] Fischer JR , Stallknecht DE , Luttrell P , Dhondt AA , Converse KA . 1997. Mycoplasmal conjunctivitis in wild songbirds: the spread of a new contagious disease in a mobile host population. Emerg Infect Dis 3:69–72. doi:10.3201/eid0301.970110 9126448 PMC2627586

[20] Ley DH , Berkhoff JE , McLaren JM . 1996. Mycoplasma gallisepticum isolated from house finches (Carpodacus Mexicanus) with conjunctivitis. Avian Dis 40:480–483. doi:10.2307/1592250 8790904

[21] Department of energy EACA. orange-bellied parrot. 2021. Victorian State Government. Available from: https://www.environment.vic.gov.au/conserving-threatened-species/threatened-species/orange-bellied-parrot

[22] Peters A , Patterson EI , Baker BGB , Holdsworth M , Sarker S , Ghorashi SA , Raidal SR . 2014. Evidence of psittacine beak and feather disease virus spillover into wild critically endangered orange-bellied parrots (Neophema chrysogaster). J Wildl Dis 50:288–296. doi:10.7589/2013-05-121 24484492

[23] Sarker S , Patterson EI , Peters A , Baker GB , Forwood JK , Ghorashi SA , Holdsworth M , Baker R , Murray N , Raidal SR . 2014. Mutability dynamics of an emergent single stranded DNA virus in a naïve host. PLoS One 9:e85370. doi:10.1371/journal.pone.0085370 24416396 PMC3885698

[24] Chang W-S , Eden J-S , Hall J , Shi M , Rose K , Holmes EC , Pfeiffer JK . 2020. Metatranscriptomic analysis of virus diversity in urban wild birds with paretic disease. J Virol 94:00606–00620. doi:10.1128/JVI.00606-20 PMC7459558 32581107

[25] Lim ES , Zhou Y , Zhao G , Bauer IK , Droit L , Ndao IM , Warner BB , Tarr PI , Wang D , Holtz LR . 2015. Early life dynamics of the human gut virome and bacterial microbiome in infants. Nat Med 21:1228–1234. doi:10.1038/nm.3950 26366711 PMC4710368

[26] Roux S , Chan L-K , Egan R , Malmstrom RR , McMahon KD , Sullivan MB . 2017. Ecogenomics of virophages and their giant virus hosts assessed through time series metagenomics. Nat Commun 8:858. doi:10.1038/s41467-017-01086-2 29021524 PMC5636890

[27] Shi M , Lin X-D , Tian J-H , Chen L-J , Chen X , Li C-X , Qin X-C , Li J , Cao J-P , Eden J-S , Buchmann J , Wang W , Xu J , Holmes EC , Zhang Y-Z . 2016. Redefining the invertebrate RNA virosphere. Nature 540:539–543. doi:10.1038/nature20167 27880757

[28] Temmam S , Monteil-Bouchard S , Robert C , Baudoin J-P , Sambou M , Aubadie-Ladrix M , Labas N , Raoult D , Mediannikov O , Desnues C . 2016. Characterization of viral communities of biting midges and identification of novel thogotovirus species and rhabdovirus genus. Viruses 8:77. doi:10.3390/v8030077 26978389 PMC4810267

[29] Zablocki O , van Zyl L , Adriaenssens EM , Rubagotti E , Tuffin M , Cary SC , Cowan D . 2014. High-level diversity of tailed phages, eukaryote-associated viruses, and virophage-like elements in the metaviromes of Antarctic soils. Appl Environ Microbiol 80:6888–6897. doi:10.1128/AEM.01525-14 25172856 PMC4249006

[30] New FN , Brito IL . 2020. What is metagenomics teaching us, and what is missed? Annu Rev Microbiol 74:117–135. doi:10.1146/annurev-micro-012520-072314 32603623

[31] Ko KKK , Chng KR , Nagarajan N . 2022. Metagenomics-enabled microbial surveillance. Nat Microbiol 7:486–496. doi:10.1038/s41564-022-01089-w 35365786

[32] Athukorala A , Phalen DN , Das A , Helbig KJ , Forwood JK , Sarker S . 2021. Genomic characterisation of a highly divergent siadenovirus (Psittacine siadenovirus F) from the critically endangered orange-bellied parrot (Neophema chrysogaster). Viruses 13:1714. doi:10.3390/v13091714 34578295 PMC8472863

[33] Tisza MJ , Buck CB . 2021. A catalog of tens of thousands of viruses from human metagenomes reveals hidden associations with chronic diseases. Proc Natl Acad Sci USA 118:e2023202118. doi:10.1073/pnas.2023202118 34083435 PMC8201803

[34] Fernández-Correa I , Truchado DA , Gomez-Lucia E , Doménech A , Pérez-Tris J , Schmidt-Chanasit J , Cadar D , Benítez L . 2019. A novel group of avian astroviruses from neotropical passerine birds broaden the diversity and host range of astroviridae. Sci Rep 9:9513. doi:10.1038/s41598-019-45889-3 31266971 PMC6606752

[35] Mollentze N , Streicker DG . 2020. Viral zoonotic risk is homogenous among taxonomic orders of mammalian and avian reservoir hosts. Proc Natl Acad Sci USA 117:9423–9430. doi:10.1073/pnas.1919176117 32284401 PMC7196766

[36] Surphlis AC , Dill-Okubo JA , Harrach B , Waltzek T , Subramaniam K . 2022. Genomic characterization of psittacine adenovirus 2, a siadenovirus identified in a moribund African grey parrot (Psittacus erithacus). Arch Virol 167:911–916. doi:10.1007/s00705-021-05341-2 35103853

[37] Harrach B , Tarján ZL , Benkő M . 2019. Adenoviruses across the animal kingdom: a walk in the zoo. FEBS Lett 593:3660–3673. doi:10.1002/1873-3468.13687 31747467

[38] Breitbart M , Delwart E , Rosario K , Segalés J , Varsani A . 2017. ICTV virus taxonomy profile: circoviridae. J Gen Virol 98:1997–1998. doi:10.1099/jgv.0.000871 28786778 PMC5656780

[39] Sarker S , Ghorashi SA , Forwood JK , Bent SJ , Peters A , Raidal SR . 2014. Phylogeny of beak and feather disease virus in Cockatoos demonstrates host generalism and multiple-variant infections within psittaciformes. Virology 460–461:72–82. doi:10.1016/j.virol.2014.04.021 25010272

[40] Sarker S , Patterson EI , Peters A , Baker GB , Forwood JK , Ghorashi SA , Holdsworth M , Baker R , Murray N , Raidal SR . 2014. Mutability dynamics of an emergent single stranded DNA virus in a naïve host. PLoS One 9:e85370. doi:10.1371/journal.pone.0085370 24416396 PMC3885698

[41] Sarker S , Ghorashi SA , Forwood JK , Bent SJ , Peters A , Raidal SR . 2014. Phylogeny of beak and feather disease virus in cockatoos demonstrates host generalism and multiple-variant infections within psittaciformes. Virology 460–461:72–82. doi:10.1016/j.virol.2014.04.021 25010272

[42] Sarker S , Ghorashi SA , Forwood JK , Raidal SR . 2013. Whole-genome sequences of two beak and feather disease viruses in the endangered swift parrot (Lathamus discolor). Genome Announc 1:e00842-13. doi:10.1128/genomeA.00842-13 24285653 PMC3860835

[43] Cotmore SF , Agbandje-McKenna M , Canuti M , Chiorini JA , Eis-Hubinger A-M , Hughes J , Mietzsch M , Modha S , Ogliastro M , Pénzes JJ , Pintel DJ , Qiu J , Soderlund-Venermo M , Tattersall P , Tijssen P , ICTV Report Consortium . 2019. ICTV virus taxonomy profile: parvoviridae. J Gen Virol 100:367–368. doi:10.1099/jgv.0.001212 30672729 PMC6537627

[44] Shan T , Yang S , Wang H , Wang H , Zhang J , Gong G , Xiao Y , Yang J , Wang X , Lu J , et al. . 2022. Virome in the cloaca of wild and breeding birds revealed a diversity of significant viruses. Microbiome 10:60. doi:10.1186/s40168-022-01246-7 35413940 PMC9001828

[45] Bankevich A , Nurk S , Antipov D , Gurevich AA , Dvorkin M , Kulikov AS , Lesin VM , Nikolenko SI , Pham S , Prjibelski AD , Pyshkin AV , Sirotkin AV , Vyahhi N , Tesler G , Alekseyev MA , Pevzner PA . 2012. SPAdes: a new genome assembly algorithm and its applications to single-cell sequencing. J Comput Biol 19:455–477. doi:10.1089/cmb.2012.0021 22506599 PMC3342519

[46] Benson DA , Clark K , Karsch-Mizrachi I , Lipman DJ , Ostell J , Sayers EW . 2014. Genbank. Nucleic Acids Res. 42:D32–7. doi:10.1093/nar/gkt1030 24217914 PMC3965104

[47] Sarker S , Talukder S , Anwar A , Van TTH , Petrovski S . 2022. Unravelling bile viromes of free-range laying chickens clinically diagnosed with spotty liver disease: emergence of many novel chaphamaparvoviruses into multiple lineages. Viruses 14:2543. doi:10.3390/v14112543 36423151 PMC9695665

[48] Sutherland M , Sarker S . 2023. Liver virome of a little corella (Cacatua sanguinea) reveals coinfection with a novel parvovirus and two beak and feather disease viruses. Aust Vet J 101:366–372. doi:10.1111/avj.13271 37497656

[49] Katoh K , Standley DM . 2013. MAFFT multiple sequence alignment software version 7: improvements in performance and usability. Mol Biol Evol 30:772–780. doi:10.1093/molbev/mst010 23329690 PMC3603318

