# Supplementary material for: Virome of Australia’s most endangered parrot in captivity evidenced of harboring hitherto unknown viruses
Source: Microbiol Spectr. 2023 Dec 4;12(1):e03052-23. doi: 10.1128/spectrum.03052-23 (PMC10783009; doi:10.1128/spectrum.03052-23)
Supplement: Supplemental File — Fig. S1-S2; Tables S1-S5. [file spectrum.03052-23-s0001.pdf]

Supplementary File

Virome of the Australia's most endangered parrot in captivity evidenced of harbouring hitherto unknown viruses.

Natalie Klukowski,[a] Paul Eden,[b] Muhammad Jasim Uddin,[c,d] and Subir Sarker[a,e*]

[a]Department of Microbiology, Anatomy, Physiology and Pharmacology, School of Life Sciences, La Trobe University, Melbourne, VIC 3086, Australia.
[b]Wildlife Conservation and Science, Zoos Victoria, Werribee, Victoria, Australia.
[c]School of Veterinary Medicine, Murdoch University, Murdoch, WA 6150, Australia.
[d]Center for Biosecurity and One Health, Harry Butler Institute, Murdoch University, Murdoch, WA 6150, Australia.
[e]Biomedical Sciences & Molecular Biology, College of Public Health, Medical and Veterinary Sciences, James Cook University, Townsville, QLD 4811, Australia.

*Address for correspondence: Dr. Subir Sarker, Biomedical Sciences & Molecular Biology, College of Public Health, Medical and Veterinary Sciences, James Cook University, Townsville, QLD 4811, Australia; email: subir.sarker@jcu.edu.au.

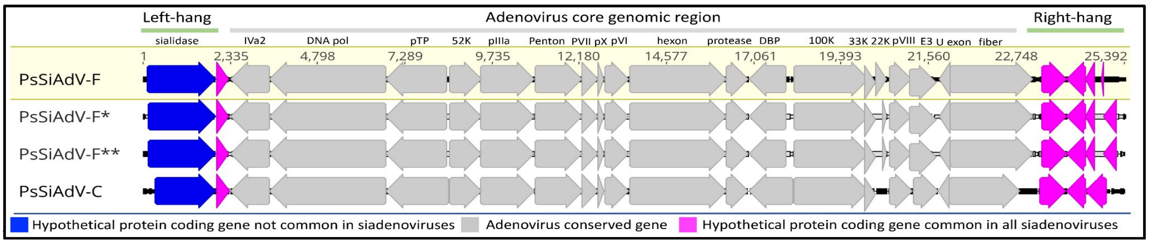

Figure S1. Schematic map of the two strains of psittacine siadenoviruses sequenced in this study, compared to two others closely related siadenoviruses found high similarity in the ORFs. (*= PsSiAdV-F, strain OBP/FE1/02/2021, GenBank accession no. OP377084; **= PsSiAdV-F, strain OBP/FE2/01/2021, GenBank accession no. OP377085). The detected viruses were compared with the previously reported PsSiAdV-F strain OBP2209 (GenBank accession no. MW365934) and PsSiAdV-C (GenBank accession no. MN687905) using Geneious Prime® (version 2022.1.1, Biomatters, New Zealand). The direction of transcription of the encoded ORFs and genes are indicated by the arrows and are coloured according to the conventions set in the legend. The open reading frames encoded within the detected viruses are highly similar and encode all 25 proteins present in the PsSiAdV-F strain OBP2209 and PsSiAdV-C.

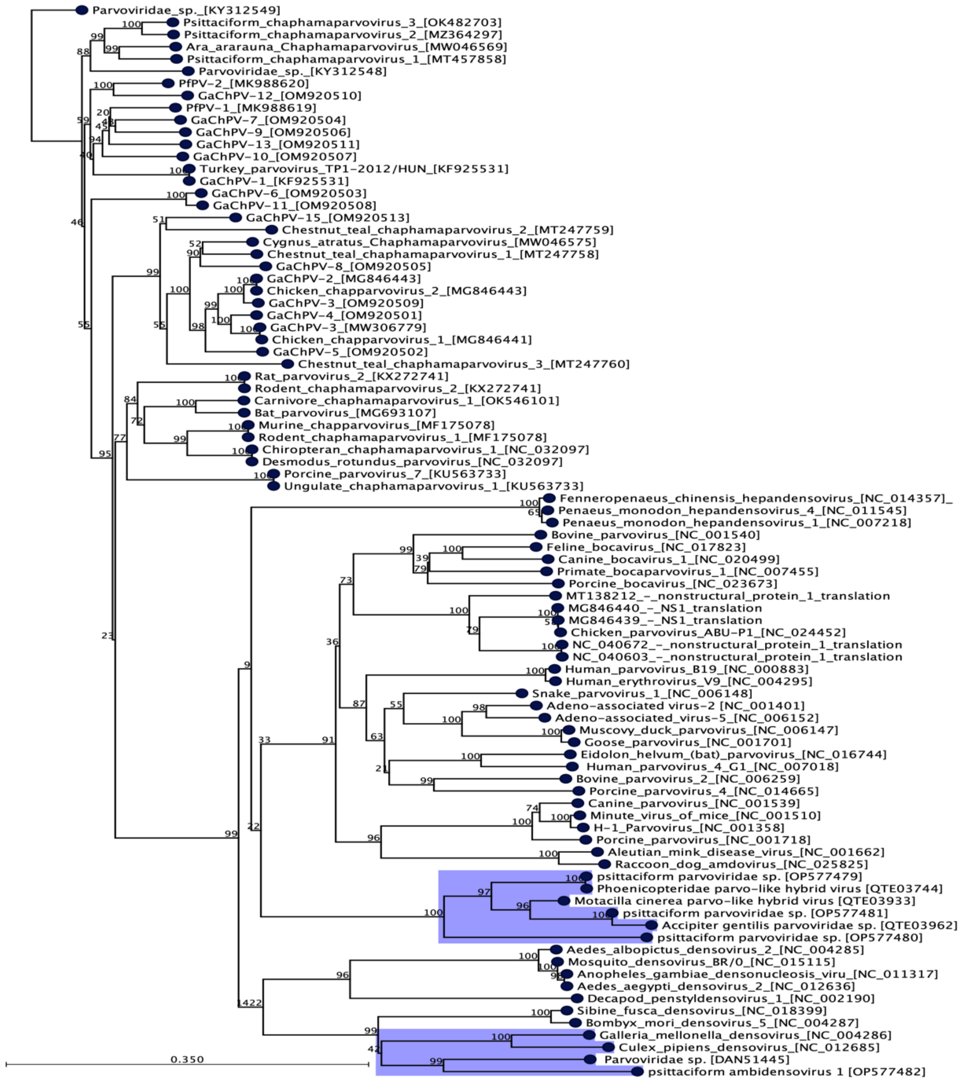

Figure S2. A maximum likelihood (ML) phylogenetic tree was generated, showing a possible evolutionary relationship of parvoviruses detected in this study (GenBank accession no. OP564879-82), with other selected parvoviruses. The tree was generated utilising selected rep-associated protein coding gene (NS1) sequences in CLC Genomic Workbench (version 9.0.1). The tree was constructed with 1000 bootstrap re-samplings. The numbers on the left show bootstrap values as percentages and the labels at branch tips refer to virus name followed by GenBank accession numbers in brackets. The parvoviruses detected within this study and related subclades are blue background.

Table S1: NGS library statistics.

Library ID	Total reads	Reads length	Total reads post-trimming	Trimmed reads (%)	Total mapped*	Total mapped reads (%)	Unmapped reads**	Unmapped reads (%)
OBP1	33,924,992	150	31,760,860	93.5	5,337,689	16.81	26,423,171	83.19
OBP2	40,686,434	150	38,133,739	93.5	7,440,207	19.51	30,693,532	80.49
OBP3	43,689,188	150	41,004,123	93.7	9,948,953	24.26	31,055,170	76.00
OBP4	33,652,490	150	31,483,885	93.4	7,387,227	23.46	24,096,658	76.54
**Mean**	**37,988,276**	**150**	**35,595,652**	**93.525**	**7,528,519**	**21.01**	**28,067,133**	**78.99**

Note: * = Number of total reads mapped to host and bacterial genomes;
** = Number of total unmapped reads that were used in de novo assembly; % = percentage

Table S2: 25 predicted protein coding genes of the two strains of PsSiAdV-F were detected in this study, with both genomes showing a 99.8% nucleotide similarity and no notable difference in the gene content.

PsSiAdV-F strains synteny (Both strains)	Genomic coordinates (nt)		Size (AA) of ORFs, both strains	Size (AA) of ORFS, PsSiAdV-F strain OBP2209	identity (%), compared to PsSiAdV-F strain OBP2209
	Strain OBP/FE1/02 /2021	Strain OBP/FE2/01 /2021			
ORF01 sialidase	118-1959	23415-25256	613	620	98.9
ORF02 hypothetical protein	1972-2301	23073-23402	109	109	100
ORF03 IVa2	2330-3418	21956-23044	362	362	100
ORF04 DNA polymerase	3408-6731	18643-21966	1107	1107	100
ORF05 pTP	6728-8461	16913-18646	577	577	100
ORF06 52K	8540-9412	15962-16834	290	290	100
ORF07 pIIIa	9402-10898	14476-15972	498	498	100
ORF08 penton	10920-12260	13114-14454	446	446	100
ORF09 pVII	12261-12650	12724-13113	129	129	100
ORF10 pX	12652-12828	12546-12722	58	58	100
ORF11 pVI	12846-13496	11878-12528	216	216	100
ORF12 hexon	13506-16274	9100-11868	922	922	100
ORF13 protease	16271-16891	8483-9103	206	206	100
ORF14 early E2	16921-17973	7401-8453	350	350	100
ORF15 100K	18178-20172	5202-7196	664	664	100
ORF16 22K	20063-20356	50185311	97	97	100
ORF17 33K	20558-20656	4718-4816	32	93	34.4

ORF18 pVIII	20717-21178	41964657	153	153	100
ORF19 E3	21126-21695	3679-4248	189	189	100
ORF20 U exon	21742-21960	3414-3632	72	72	100
ORF21 fibre protein	21959-22990	2384-3415	343	343	100
ORF22 hypothetical protein	23107-23784	1590-2267	225	225	100
ORF23 hypothetical protein	23795-24319	1055-1579	174	174	100
ORF24 hypothetical protein	24319-24567	807-1055	82	82	100
ORF25 hypothetical protein	24835-25185	189-539	116	175	66.3

Table S3: Predicted protein coding genes of circoviruses and CRESS-DNA viruses

**Crucivirus strain OBP1/FE1/23/2022 detected in OBP1 (GenBank accession no. OP564891)**							
**Product synteny**	**Genome coordinates**	**Best match, accession no.**	**Size (AA)**	**Reference size (AA)**	**%. identity**	**e-value**	**Query cov (%)**
ORF01 capsid protein	845-2350	QYW06880	501	194	96.91	1×10^{-129}	38
ORF02 rep-associated protein	2428-3894	QMW68946	488	530	28.57	3×10^{-31}	75
ORF03 hypothetical protein	691-32		219				
**Tick-associated circular DNA virus strain OBP1/FE1/85/2022 detected in OBP1 (GenBank accession no. OP564893)**							
**Tick-associated circovirus synteny**	**Genomic coordinates**	**Best match, accession no.**	**Size (AA)**	**Reference size (AA)**	**%. identity**	**e-value**	**Query cov (%)**
ORF01 rep-associated protein	4-720	UTM74954	238	675	100	7×10^{-170}	100
ORF02 capsid protein	754-1518	UTM74955	254	765	100	4×10^{-168}	100

ORF03 hypothetical protein	22-243		73				
**CRESS virus sp. strain OBP1/FE1/74/2022 detected in OBP1 (GenBank accession no. OP564892)**							
**CRESS virus synteny**	**Genomic coordinates**	**Best match, accession no.**	**Size (AA)**	**Reference size (AA)**	**%. identity**	**e-value**	**Query cov (%)**
ORF01 capsid protein	379-1254	QJI53689	291	274	26.74	1×10^{-13}	86
ORF02 rep-associated protein	1976-1251	QSX73072	241	302	44.53	1×10^{-58}	99
ORF03 hypothetical protein	115-2446		43				

Table S4: Predicted protein coding genes of parvoviruses detected in this study

**Psittaciform parvoviridae sp. OBP1/FE1/44/2022 (GenBank accession no. OP577481)**							
**Parvovirus synteny**	**Genomic coordinates**	**Best match, accession no.**	**Size (AA)**	**Reference size (AA)**	**%. identity**	**e-value**	**Query cov (%)**
ORF01 hypothetical protein	2712-2476		78				
ORF02 replication protein	2343-940	QTE03933	467	411	67.77	0.0	83%
ORF03 capsid protein	927-517	QDI06016	136	679	39.77	2×10^{-08}	64
**Psittaciform parvoviridae sp. strain OBP1/FE1/49/2022 (GenBank accession no. OP577480), detected in OBP1**							
**Parvovirus synteny**	**Genomic coordinates**	**Best match, accession no.**	**Size (AA)**	**Reference size (AA)**	**%. identity**	**e-value**	**Query cov (%)**
ORF01 rep-associated protein	304-1509	QTE03962	401	394	28.82	9×10^{-36}	97
ORF02 hypothetical protein	2561-1470		363				
**Psittaciform parvoviridae sp. strain OBP1/FE1/29/2022 (GenBank accession no. OP577479), detected in OBP1**							

Parvo-hybrid virus synteny	Genomic coordinates	Best match, accession no.	Size (AA)	Reference size (AA)	%. identity	e-value	Query cov (%)
ORF01 replication protein	2679-1513	QTE03744.1	388	388	99.23	0.0	100
ORF02 hypothetical protein	1494-1	QTE03745.1	497	503	92.57	0.0	100
**Psittaciform ambidensovirus 1 strain OBP4/DE2/13/2022 (GenBank accession no. OP577482), detected in OBP4**							
**Partial parvovirus genome synteny**	**Genomic coordinates**	**Best match, accession no.**	**Size (AA)**	**Reference size (AA)**	**%. identity**	**e-value**	**Query cov (%)**
ORF01 capsid protein	571-2343	QVW56842	590	444	43.13	1×10^{-89}	69
ORF02 NS1 replication protein	3916-2804	DAN51445	370	577	30.56	5×10^{-50}	97
ORF02 NS2 replication protein	4074-3208	QVW56849.1	288	229	25.77	2×10^{-04}	55

Table S5: Predicted protein coding genes of picornaviruses detected in this study

**Psittacine picornavirus 1 strain OBP4/DE2/3/2022 (OP577483), detected in OBP4**							
**Picornavirus genome synteny**	**Genomic coordinates**	**Best match, accession no.**	**Size (AA)**	**Reference size (AA)**	**%. identity**	**e-value**	**Query cov (%)**
ORF01 RdRP hypothetical protein	1436-6544	YP_00933346 1	1702	2368	29.77	$2 \times 10\text{-}^{36}$	40
ORF02 hypothetical protein	601-1089		162				
**Psittacine picornavirus 2 strain OBP4/DE2/9/2022 (GenBank accession no. OP577484), detected in OBP4**							
**Picornavirus genome synteny**	**Genomic coordinates**	**Best match, accession no.**	**Size (AA)**	**Reference size (AA)**	**%. identity**	**e-value**	**Query cov (%)**
ORF01 hypothetical protein	2214-1594		205				
ORF02 hypothetical protein	3915-2257	YP_00934225 5	551	535	26.46	6×10^{-27}	85

ORF03 Hypothetical protein	4833-3887	DAZ87942	314	1966	34.08	1×10^{-20}	65
